# Prática da enfermeira na atenção primária à saúde em municípios rurais remotos

**DOI:** 10.1590/0102-311XPT208124

**Published:** 2025-10-03

**Authors:** Adriano Maia dos Santos, Lígia Giovanella, Cassiano Mendes Franco, Aline Gonçalves Pereira, Juliana Gagno Lima, Márcia Cristina Rodrigues Fausto, Patty Fidelis de Almeida

**Affiliations:** 1 Instituto Multidisciplinar em Saúde, Universidade Federal da Bahia, Vitória da Conquista, Brasil.; 2 Escola Nacional de Saúde Pública Sergio Arouca, Fundação Oswaldo Cruz, Rio de Janeiro, Brasil.; 3 Faculdade de Medicina, Universidade Federal do Rio de Janeiro, Rio de Janeiro, Brasil.; 4 Instituto de Saúde Coletiva, Universidade Federal do Oeste do Pará, Santarém, Brasil.; 5 Instituto de Saúde Coletiva, Universidade Federal Fluminense, Niterói, Brasil.

**Keywords:** Enfermagem, Serviços de Saúde Rural, Atenção Primária à Saúde, Acesso aos Serviços de Saúde, Padrões de Práticas de Enfermagem, Nursing, Rural Health Services, Primary Health Care, Health Services Accessibility, Nurses’ Practice Patterns, Enfermería, Servicios de Salud Rural, Atención Primaria de Salud, Acceso a los Servicios de Salud, Pautas de la Práctica en Enfermería

## Abstract

Analisam-se as práticas desenvolvidas por enfermeiras em equipes de saúde da família (EqSF) de municípios rurais remotos. Trata-se de um estudo de casos múltiplos, com abordagem qualitativa, realizado por meio de 52 entrevistas semiestruturadas com enfermeiras de 27 municípios rurais remotos distribuídas entre 10 estados. De acordo com os resultados, as enfermeiras destacaram-se, com poucas especificidades entre os municípios rurais remotos, por desenvolverem um escopo variado de competências em atividades gerenciais, práticas assistenciais individuais e ações no território. Independentemente dos obstáculos impostos ao atendimento das EqSF em todos os municípios rurais remotos, sobressaiu-se a articulação das enfermeiras na busca por contornar as adversidades, restabelecer fluxos para os usuários e minimizar ao máximo a desassistência. As enfermeiras, em todos os municípios rurais remotos, buscavam restabelecer o vínculo comunicacional e viabilizar a continuidade do cuidado das pessoas que necessitavam de alguma assistência fora da atenção primária à saúde (APS). De forma unânime, nenhuma enfermeira estabelecia uma interlocução entre o saber popular e as ações biomédicas das práticas consolidadas na APS, ainda que reconhecessem a diversidade cultural e étnica que povoava os territórios. Em síntese, as enfermeiras nos municípios rurais remotos buscavam driblar as adversidades comunicacionais e forjavam, ainda que limitadas, práticas sensíveis às demandas das pessoas e da comunidade.

## Abstract (en)

Practices developed by nurses of family health teams (FHTs) in remote rural municipalities were analyzed. A multiple case study with a qualitative approach was conducted through 52 interviews with nurses from 27 remote rural municipalities distributed across 10 states. With few specificities among the remote rural municipalities, nurses stood out for developing a broad range of skills related to management activities, individual care practices, and actions in the local community. Irrespective of the obstacles to care faced by FHTs, the work of nurses stood out in all remote rural municipalities to overcome adversities, reestablish patient flow, and minimize the lack of care as much as possible. In all remote rural municipalities, nurses sought to reestablish the communication link and enable the continuity of care for individuals who required assistance outside primary care. None of the nurses established dialogue between folk knowledge and the biomedical actions of the practices established in primary care but recognized the cultural and ethnic diversity in the local communities. In summary, nurses in the remote rural municipalities sought to overcome communication difficulties and forged - although in a limited way - practices sensitive to the needs of individuals and the community.

## Abstract (es)

Se analizan las prácticas desarrolladas por los profesionales de enfermería en equipos de salud familiar (EqSF) en municipios rurales remotos. Se trata de un estudio de caso múltiple, con enfoque cualitativo, realizado mediante la aplicación de 52 entrevistas semiestructuradas con los profesionales de enfermería de 27 municipios rurales remotos distribuidos en 10 estados brasileños. Los resultados revelan que los profesionales de la enfermería se destacaron, con pocas especificidades entre los municipios rurales remotos, por desarrollar una amplia gama de competencias en actividades gerenciales, en prácticas de cuidado individual y en acciones en el territorio. Independientemente de los obstáculos impuestos a la atención en las EqSF en todos los municipios rurales remotos, la articulación de estos profesionales se destacó en la búsqueda de superar las adversidades, de restablecer los flujos para los usuarios y de minimizar al máximo la falta de atención. Los profesionales de enfermería en todos los municipios rurales remotos buscaron restablecer el vínculo de comunicación y permitir la continuidad de la atención a las personas que necesitaban asistencia fuera de la atención primaria de salud (APS). Por unanimidad, ninguno de ellos estableció un diálogo entre el conocimiento popular y las acciones biomédicas de las prácticas establecidas en la APS, a pesar de reconocer la diversidad cultural y étnica de los territorios. Se concluye que estos profesionales en los municipios rurales remotos buscaron eludir las adversidades de la comunicación y trazaron prácticas sensibles, aunque limitadas, a las demandas de las personas y la comunidad.

## Introdução

A atração e fixação de enfermeiras para atuarem na atenção primária à saúde (APS) em áreas rurais e remotas permanece um grande desafio em escala global ^1^, seja em países de alta ^2^, média ^3^ ou baixa renda ^4^. As especificidades do trabalho em contextos de ruralidade − insuficiência de transporte e trafegabilidade precária ^5^, populações dispersas e condições socioeconômicas historicamente desfavoráveis ^6^, vazios assistenciais ^7^, pior infraestrutura e acesso aos serviços de saúde em tempo oportuno ^8^, entre outros − tencionam para que enfermeiras desenvolvam competências assistenciais ^9^ ^,^ ^10^ e gerenciais ^11^ em um escopo ampliado de práticas ^12^, além de forte atuação comunitária ^13^. 

Não obstante, estresse, isolamento social, geográfico ou profissional influenciam o trabalho de enfermeiras em contexto rural e, ao mesmo tempo, condicionam o desenvolvimento de práticas isoladas, com pouco apoio de outros profissionais, em cenários que vão de unidades de APS à regulação assistencial de urgência e emergência ^14^ ^,^ ^15^. O próprio tempo gasto com o deslocamento interfere na viabilização da assistência em meio rural ^5^, na medida em que diminui a permanência dos profissionais nas unidades ^16^, exigindo um conjunto de adequações nos processos de trabalho de toda a equipe de APS ^17^ ^,^ ^18^.

A competência cultural ^13^ é um atributo fundamental nos territórios rurais, presente na atuação da enfermeira e necessário à oferta de cuidados integrais que, rotineiramente, extrapolam as atividades assistenciais ^19^. O alinhamento entre saberes tradicionais e a prática da enfermeira em contexto rural inclui conexões pessoais e profissionais com a comunidade local, sensação de pertencimento, amplo escopo de práticas e atuação que, comumente, extrapola o período laboral oficial ^20^. Por conseguinte, conhecer como as enfermeiras organizam seu processo de trabalho pode colaborar para a implementação de políticas e ações para o enfrentamento dos problemas identificados, contribuir para produção do cuidado aos usuários e influenciar positivamente na satisfação com o trabalho em cenário rural ^21^. 

Para tanto, compreende-se que o processo de trabalho da enfermeira ^22^ envolve a interface entre a dimensão gerencial ^11^ e a dimensão do cuidado ^23^, muito embora a precarização do trabalho de enfermeiras ^24^ ^,^ ^25^ e a formação hegemônica ^26^ mitiguem a potência da clínica ampliada ^27^ e do trabalho interprofissional ^28^. Deste modo, analisam-se as práticas desenvolvidas por enfermeiras em equipes de saúde da família (EqSF) de municípios rurais remotos.

## Metodologia

Este artigo analisa parte dos resultados da pesquisa *Atenção Primária à Saúde em Municípios Rurais Remotos no Brasil* ^29^. Partiu-se da caracterização dos 323 municípios rurais remotos, segundo classificação do Instituto Brasileiro de Geografia e Estatística (IBGE) ^30^, que foram tipificados segundo características socioeconômicas, demográficas e de saúde e definidos seis *clusters* com lógicas espaciais específicas: Matopiba, Norte Minas, Norte Águas, Norte Estradas, Semiárido e Vetor Centro-Oeste ^7^. 

A amostra de municípios foi intencional, estruturada a partir dos seis *clusters*, nos quais foram escolhidos dois ou mais municípios que corresponderiam ao “município tipo”, ou seja, com características socioeconômicas, demográficas e de saúde mais frequentes no conjunto dos municípios rurais remotos do respectivo *cluster*. Ao “município tipo”, agregou-se um ou mais municípios *outliers*. Ao final, foram selecionados 27 municípios rurais remotos distribuídos em 10 estados − Acre, Amapá, Amazonas, Bahia, Maranhão, Mato Grosso, Minas Gerais, Pará, Piauí e Tocantins − para realização de estudo de casos múltiplos, com abordagem qualitativa, por meio de entrevistas semiestruturadas ^29^. 

Em cada município rural remoto, foram entrevistadas 52 enfermeiras de unidades básicas de saúde (UBS) localizadas na zona urbana e na zona rural, para compreensão do processo de trabalho e compartilhamento de práticas na APS. As entrevistas foram codificadas para garantir o anonimato. 

As entrevistas foram presenciais, realizadas nos respectivos locais de trabalho, entre maio a outubro de 2019, com duração média de 60 minutos, gravadas em áudio e transcritas na íntegra. Os roteiros semiestruturados, disponíveis no site da pesquisa (https://apsmrr.ensp.fiocruz.br/wp-content/uploads/2021/10/ROTEIRO-PROFISSIONAIS.pdf), serviram de apoio ao entrevistador, sendo as enfermeiras estimuladas a responder aos temas abordados de forma espontânea.

Este artigo inspira-se na compreensão do processo de trabalho em saúde na perspectiva de Mendes-Gonçalves ^31^ e leva em consideração a interface entre a dimensão gerencial e a dimensão do cuidado na prática da enfermeira exercida em realidades rurais. Para tanto, a interpretação dos dados foi orientada pela análise de “conteúdo temática”, identificando-se convergências e divergências. Realizou-se a ordenação dos dados a partir da leitura geral do material transcrito. O *corpus* foi ordenado e classificado, etapas em que as transcrições foram lidas exaustivamente e assim emergiram 34 componentes temáticos (Quadros 1, 2, 3 e 4). 

**Quadro 1:** Protagonismo gerencial das enfermeiras nos territórios: porta de entrada e organização da agenda.

COMPONENTE TEMÁTICO	EXTRATOS DE FALAS DOS ENTREVISTADOS
Corriqueiramente a UBS era o primeiro ponto de contato da população na rede de atenção	“*Ele* [usuário] *primeiro costuma vir direto à unidade ou, às vezes, ele fala com o ACS”* (Bonito de Minas, Minas Gerais, Norte Minas)
	“*Ele* [usuário] *vem primeiro aqui. Podem tá passando mal, até pra parir; eles passam primeiro aqui, pra eu dizer que tem que ir pra lá* [hospital]. (...) *Até pra ir ao [serviço] particular, eles passam aqui pra dizer que vão ao particular*” (Ipupiara, Bahia, Semiárido)
	“*Sempre a UBS* [é o primeiro ponto de contato], *porque é mais perto e devido a carência da população*” (Vitória do Jari, Amapá, Norte Águas)
UBS funcionavam em horário comercial com poucas variações	“*A gente inicia às 8 horas, o atendimento, fecha meio-dia, retorna às 14 horas e fecha às 17h*. (...) *De segunda a sexta e, às vezes, o dentista atende no sábado, na parte da manhã*” (Ipupiara, Bahia, Semiárido)
	“*De segunda-feira à sexta-feira, das 7h30 às 11h30 e das 13h30 às 17h30*” (Vitória do Jari, Amapá, Norte Águas)
	“[o funcionamento da UBS] *é das 7 às 11h e das 13 às 17h*” (Vila Bela da Santíssima Trindade, Mato Grosso, Vetor Centro-Oeste)
	“*Das 8h às 17h, de segunda à sexta, tem o intervalo do almoço e volta*” (Júlio Borges, Piauí, Matopiba)
	“*O nosso horário é das 7h30min até 11h30min e, à tarde, funciona das 13h30min até 17h30min*” (Jacareacanga, Pará, Norte Estradas)
	“*Segunda à sexta, de 7h às 11h e de 13h às 17h, isso para o técnico e demais funcionários. O médico faz horário corrido*” (Rubelita, Minas Gerais, Norte Minas)
Organização dos fluxos administrativos e assistenciais, bem como das práticas na comunidade, centrados nos enfermeiros	“*Nesse momento não está tendo* [atividade educativa], *como estou sozinha na unidade, tenho que suprir com toda a demanda e é muita coisa só para um enfermeiro*” (Jacareacanga, Pará, Norte Águas)
	“*Hoje, sou enfermeira da unidade e gestora da unidade. Coordenadora e enfermeira, então, a gente está em duas funções em uma. Que deveria ser só o horário comercial,* [mas] *trabalho em tempo integral. Não vou dizer que concordo, mas me acostumei*” (Morpará, Bahia, Semiárido)
	“*Eu que levo, que encaminho* [as solicitações]. *O médico faz, às vezes. Ele até me chama na sala e fala: ‘oh, pra esse atendimento aqui, tenho uma maior necessidade’. Aí, ele pede pra escrever ‘urgente’, ligo para o pessoal que faz essa marcação e aviso que o médico pediu para ser o mais rápido possível* (...). *Fica tudo comigo, coloco em um envelope e o motorista leva. Eu ainda ligo e oriento direitinho o que é que tem que ser feito*” (Rubelita, Minas Gerais, Norte Minas)
Agenda era organizada, predominantemente, por ações programáticas por ciclo de vida ou condição de saúde	“*A gente trabalha com cronograma semanal: segunda é criança, então é só pra criança; quarta é só gestante, mas nada disso atrapalha de a gente atender a demanda espontânea. A gente segue o cronograma, mas junto a demanda espontânea; um dia pra teste rápido*” (Vitória do Jari, Amapá, Norte Águas)
	“*Dia de quarta, é gestante; dia de quinta; é o dia do hipertenso e aqueles que usam medicamento controlado, saúde mental e aqueles que só precisam e fazem acompanhamento e que vai usar medicação por 6 meses, que só tem que fazer a troca de receita, aí, eles vêm na quinta, a gente faz a troca da receita. Na segunda e na sexta, é demanda* [livre]. *Na quinta-feira à tarde, é feito teste rápido, mas se tiver alguma emergência, a gente faz também*” (Nova Lacerda, Mato Grosso, Vetor Centro-Oeste)
	“*A gente tem um cronograma, segunda de manhã, por exemplo, faz pré-natal, e o médico faz consulta agendada e consulta normal. Segunda à tarde, a gente faz também consulta, aí, vai indo; terça, visita, eu vou com ACS; à tarde, tem o dia dos idosos; aí, na quarta-feira pela manhã, a gente faz o hiperdia; na quinta de manhã, eu faço prevenção e ele tá fazendo consulta normal; aí, na quinta à tarde, ou é atendimento domiciliar ou pré-natal, e, na sexta, a gente vai se virando*” (Júlio Borges, Piauí, Matopiba)
Equipes com menor flexibilidade na organização da oferta de serviços e focadas em ações programáticas	“*Na segunda, é teste do pezinho; na terça, é inscrição pré-natal; na quarta, é seguimento; na quinta, é preventivo; e na sexta, é demanda espontânea*” (Jacareacanga, Pará, Norte Estradas)
Equipes cujo atendimento, via demanda espontânea, era o padrão prevalente ou mesmo a única forma de oferta de serviços	“[a organização da agenda] *é por demanda espontânea, a gente não está conseguindo trabalhar, ainda, com agendamento. A gente atende a todo mundo que chega*” (Tabaporã, Mato Grosso, Vetor Centro-Oeste)
	“...*as fichas de dente são distribuídas pelos ACS, as consultas programadas pelos ACS, mas a gente ainda tem 80% da população que vem de demanda espontânea pra UBS*” (Indaiabira, Minas Gerais, Norte Minas)
	“*É mais espontânea! Eu acho que é mais espontânea. Porque quando a gente está tentando organizar a agenda, aí, tem uma mudança, por exemplo, um médico. Deu aquele problema dos cubanos do Programa Mais Médicos. Quando eram os Médicos brasileiros, passavam só uma semana aqui e iam embora, os que têm CRM, iam embora. Aí a gente ficava sem Médico. Então, não tem como fazer uma agenda*” (Boa Vista de Ramos, Amazonas, Norte Águas)
Equipes com atendimento balanceado entre demanda espontânea e organizada	“*Quando é* [demanda espontânea] *da zona rural, sempre olho com um carinho diferente; agora, quando é uma pessoa que mora perto do posto, aí, converso e peço para voltar no dia certo mesmo*” (Assis Brasil, Acre, Norte Estradas)
	“*Temos agenda* [organizada] *da médica, enfermeira e dentista; aí, a gente acolhe, se a gente tiver vaga, encaixa no mesmo dia, se não marca pra amanhã* (...). *Hoje, por exemplo, a doutora deve ter atendido quase 40 pessoas, por que assim, a gente atende pra não fazer voltar, tenta resolver o problema naquele momento que a pessoa chegou.* (...) *e se tiver muito cheio no dia, aí, ela agenda pro outro dia, mas se tiver vaga no mesmo dia, é atendido, no mesmo dia*” (Ipupiara, Bahia, Semiárido)
Equipes sensíveis às peculiaridades de populações da zona rural, particularmente as que residiam em territórios mais longínquos ou com maior dificuldade de transporte	“*Então, a zona rural, eles* [usuários] *têm muita dificuldade de transporte pra vir pra cá* [zona urbana]. *Então, não é todo dia que acham transporte pra vir pra cá. O dia que tem transporte é o dia da feira, que é segunda-feira, aí, a segunda-feira já é a demanda pra zona rural; pro da sede, a gente pode marcar pra manhã ou dependendo pro turno da tarde*” (Ipupiara, Bahia, Semiárido)
	“*Das comunidades, por exemplo, São José, Mamãe Anã, Boca do Limão, Porto Rico e os garimpos. Não é nosso, mas a gente não deixa de atender, porque não podemos negar atendimento.* (...). *Eles vêm da beira do rio*” (Jacareacanga, Pará, Norte Estradas)
Atendimento era itinerante, com visitas esporádicas da equipe a diferentes comunidades e atendimento em locais adaptados	“*Viajamos de segunda à quinta-feira, temos um cronograma* [cada dia a equipe vai em uma comunidade]. *A gente marca,* (...) *pra atender hipertenso, diabético, vacina e visita domiciliar. Então, em um dia a gente faz tudo isso. O médico também, ele vai, tem os dias do médico ir, no caso desse mês, como estamos trabalhando na prevenção do câncer de mama, colo de útero, então eu fiz aqui pra atender todas as minhas comunidades pra fazer o Papanicolau*” (Bonito de Minas, Minas Gerais, Norte Minas)
	“*A minha saída geralmente é na terça-feira de Vitória do Jari* [sede]. *Minha primeira parada é comunidade Paga Dívida, tem umas 7 casas e um posto de saúde, aí, eu faço atendimento, procuro levar a vacina e passo adiante. Já subo* [pelo rio], *venho fazendo Marajó, mesmo procedimento, distribuo a medicação, faço atendimento, levo atendimento odontológico* [apenas extração dentária] *e a vacina. Por fim, chegamos aqui na UBS de Jarulândia, onde temos um atendimento maior*” (Vitória do Jari, Amapá, Norte Águas)
Demanda era organizada pelos ACS ou, na ausência desses, por algum líder comunitário	“...*suponha-se que em um ano, nós passamos duas vezes em cada comunidade, depende muito da demanda. Por exemplo, se o responsável* [líder comunitário] *pela comunidade achar que está precisando, eles entram em contato com a secretaria; a secretaria faz uma análise e se for para ir, nós vamos*” (Jacareacanga, Pará, Norte Estradas)
	“...*através do ACS, eles vêm, trazem o caso e a gente vai lá* [na comunidade] *fazer as ações; tudo é através do ACS*” (Rurópolis, Pará, Norte Estradas)
Áreas descobertas influenciavam negativamente na oferta de uma agenda programática	“A *gente atua fazendo mutirão. Somente nos programas específicos dos hipertensos, diabéticos, grávidas e nas crianças. Não é com muita frequência. Como a área de abrangência é muito grande e eu tenho duas microáreas descobertas. Faço uma vez no mês, é o que tenho feito*. (...) *Então, uma vez no mês, vamos* [a equipe] *visitar aquela área*” (Maués, Amazonas, Norte Águas)
Ciclo das águas determina o fluxo dos usuários nas UBS	“...*no inverno, nós atendemos ribeirinhos e, no verão, o pessoal das estradas*” (Enfermeiro 1, Assis Brasil, Acre, Norte Estradas)
	“*No verão, demoram* [usuários] *umas quatro horas de viagem* [até a UBS] *e no inverno é mais rápido. Eles vêm de voadeira*” (Jacareacanga, Pará, Norte Águas)
	“*Nesse período, agora, está melhorando de transição dos usuários. No inverno é o problema, dá para vir pela estrada, ônibus, carro e moto, mas quando o rio está cheio o acesse é feito pelo rio, depois de secar o rio é só por estrada; o usuário vai ter que fazer um novo caminho para chegar aqui* [na UBS] *de novo, então, querendo ou não, fica complicado de acessar por causa das chuvas*” (Curuá, Pará, Norte Águas)
Sobressaiu-se a articulação das enfermeiras na busca por contornar as adversidades, reestabelecer fluxos para os usuários e minimizar ao máximo a desassistência	“...*já teve caso de deixarmos o carro na estrada e ir andando até a casa da paciente. Às vezes, a gente tem que ir buscar grávida que está com dor para parir na vicinal; já fui de moto, às vezes, a gente vai fazer vacinação de moto que é mais fácil de chegar nas comunidades, mas a gente consegue chegar*” (Rurópolis, Pará, Norte Estradas)
	“*Às vezes, quando está sem médico, teve uns três meses sem médico no município, a gente atendia com a consulta de enfermagem. Os casos mais graves, a gente encaminhava para o hospital, que é o único lugar que não fica sem médico.* (...) *essa rotatividade afeta o trabalho da enfermagem, porque sobrecarrega o profissional. A gente fica tipo, fazendo trabalho em dobro*” (Boa Vista de Ramos, Amazonas, Norte Águas)
Havia enfermeiras que residiam na zona rural e ficam à disposição para atender/encaminhar as intercorrências	“...*moro bem aqui, na outra esquina* [próximo à UBS rural], *então, quando elas* [técnicas de plantão] *não dão conta de resolver aquele caso mais complexo, chama a gente* [à noite]. (...). *Como te falei, todo mundo conhece todo mundo, às vezes, a pessoa nem vem no posto, ela vai na minha casa: ‘Enfermeira, vamos lá, fulano está assim, assim, assim!’. A gente tem o coração mole e tem que ir, acabo vindo, às vezes, 3 horas da manhã*” (Rurópolis, Pará, Norte Estradas)
	“*Eu fico aqui, mas eles me dão todos os subsídios para que eu esteja morando aqui na zona rural - local, almoço, isso é por conta da secretaria*” (Morpará, Bahia, Semiárido)

ACS: agentes comunitários de saúde; CRM: Conselho Regional de Medicina; UBS: unidades básicas de saúde.

**Quadro 2:** Abrangência e longitudinalidade das práticas da enfermeira: ações compartimentalizadas e centradas na queixa-conduta.

COMPONENTE TEMÁTICO	EXTRATOS DE FALAS DOS ENTREVISTADOS
Equipes desfalcadas geravam demandas acumuladas	“*Aumenta a demanda,* [pois] *preciso atender os meus e, também, dá conta dos* [pacientes] *deles* [médicos] (...). *Eu passei quase 6 meses sem médico!!!* (...) [para cuidar] *você tem que conhecer a população,* (...), *quando você fica nessa rotatividade, não consegue conhecer a população que você atende, então, você não atende da maneira específica pra’quela população*” (Ipupiara, Bahia, Semiárido)
	“*Interfere* [falta de médico], *porque a gente fica um pouco sobrecarregada. A gente tem que atender de tudo e fazer de tudo, então, não tenho um tempo assim bem específico. Eu já tento dividir, porque já consegui controlar agora a população, então já consigo ter um horário para gestantes, crianças e pra idosos, mas antes ficava bem tumultuado para fazer o atendimento geral*” (Vitória do Jari, Amapá, Norte Águas)
	“*Porque antes trabalhávamos por agendamento, a pessoa vinha e agendava a sua consulta, mas como ficamos muito tempo sem médico* [6 meses] *e os profissionais passaram a ficar poucos, só estávamos com um médico, em praticamente todo o município, passamos para a dispensação de fichas, as pessoas vinham para pegar uma vaga* (...). *A demanda lota*” (Monte Alegre, Piauí, Matopiba)
Práticas isoladas e compartimentalizadas ou com colaboração pontual	“*Temos na agenda, um dia na semana para cada: para o médico e para o enfermeiro. Na quinta, é o dia da doutora; ela vai atender hipertenso e diabético. Na terça, eu que atendo o hipertenso e diabético. Assim como para outras, tipo, pré-natal, entendeu?*” (Boa Vista de Ramos, Amazonas, Norte Águas)
	“*Na coleta* [Papanicolau], *se eu identifico alguma anomalia, chamo logo o médico para dar uma avaliada e ele vai trabalhar em cima disso* (...)” (Curuá, Pará, Norte Águas)
	“*As nossas agendas funcionam de formas diferentes para o médico, o enfermeiro e o dentista. Para o enfermeiro tudo funciona pelos programas e cada programa tem o seu dia.* (...) *Se eu identifiquei* [uma grávida], *já encaminho para o médico para que ele, também, faça a sua avaliação*” (Maués, Amazonas, Norte Estradas)
Atendimento intercalado entre médico e enfermeiro com revezamento na conduta clínica	“...*atendimentos de puericultura, atendimentos dos grupos de risco sempre são compartilhados* [intercalado] *conosco* [médico e enfermeira]” (Monte Alegre, Piauí, Matopiba)
	“*Ele* [médico] *faz e eu faço* [consulta de pré-natal]. A *gente tenta alternar as consultas, quando não consegue fazer uma comigo, faz duas comigo e duas com ele, a gente faz tanto de pré-natal quanto de puericultura*” (Morpará, Bahia, Semiárido)
Trabalho da enfermagem (apoio) subordinado à prática médica	“*A parte do próprio atendimento do médico, eu faço toda a classificação de risco para o médico e, caso ele precise da minha ajuda, eu vou estar ali para ajudar, tipo para avaliar o paciente*” (Jacareacanga, Pará, Norte Estradas)
Enfermeiro realizava as ações programáticas e assumia as práticas preteridas pelos médicos	“*A gente faz a coleta* [Papanicolau], *mais os enfermeiros, mas o médico, às vezes coleta, não é? É mais assim, educação em saúde e a coleta do preventivo* [é a enfermeira]. *Quando vem o resultado, o laudo, o médico faz o encaminhamento*” (Boa Vista de Ramos, Amazonas, Norte Águas)
	“*Sobre a prevenção do colo do útero, atualmente, sou eu que coleto*” (zona rural, Morpará, Pará, Semiárido)
Casos crônicos controlados ficavam sob responsabilidade do enfermeiro e os casos descompensados eram direcionados ao médico	“*Divide* [as práticas], *a gente coloca os descompensados tudo para ele* [médico]. *Aí, os que estão com a pressão controlada passam comigo e com a outra enfermeira* [outra equipe]; *já os descompensados, diabéticos e hipertensos, vão para o médico* (...), *a gente divide assim, os prontuários*” (Rurópolis, Pará, Norte Estradas)
Embora menos comum, havia consultas sequenciais no mesmo dia entre médico e enfermeiro ou, ainda, atendimento conjunto para alguns casos clínicos	“*A maioria, gestante, o dia de hiperdia, bastante* [consulta compartilhada]. (...) *De acordo o caso, chamo* [a médica] *aqui; vou lá na sala dela e peço: ‘doutora, dá um pulinho aqui na minha sala’. Aí, ela vem aqui na minha sala, se precisar prescrever ou encaminhar pra alguma especialidade, ela já faz isso*” (Ipupiara, Bahia, Semiárido)
	“*Às vezes, tem uma grávida que está com hipertensão, a gente senta e vamos discutir o caso dela, fazer um estudo. Sempre com aquelas que dão mais problemas, a gente tenta sempre fazer um estudo sobre elas, compartilhar*” (Boa Vista de Ramos, Amazonas, Norte Águas)
Reuniões de equipe convocadas e presididas pelos enfermeiros	“[Sobre as reuniões de equipe] *Os ACS, as técnicas, eu* [participamos], *o médico não participa muito, o odontólogo está sempre ocupado*” (Jacareacanga, Pará, Norte Estradas)
Trabalho compartilhado restringia-se às ações de educação em saúde na comunidade	“*Geralmente temos grupos que a gente monta, alguns com hipertensão, outros com diabetes. Temos gestantes, gravidez na adolescência, incluímos muitos psicólogos, assistente social. Temos esse problema com abuso de álcool porque aqui não tem outra coisa pra fazer, o povo bebe demais. Dessa forma, a gente monta muitos grupos*” (Tasso Fragoso, Maranhão, Matopiba)
	“*Na minha área tem uma creche, todo ano eu vou, faço as medidas antropométricas, avalio junto com a nutricionista* (...). *O dentista, também, vai fazer avaliação bucal nas crianças, na escola. Na escola, a gente trabalha com higiene, o dentista trabalha essa parte de higiene bucal, as meninas do NASF, também, vão*” (Ipupiara, Bahia, Semiárido)

ACS: agentes comunitários de saúde; NASF: Núcleo de Apoio à Saúde da Família.

**Quadro 3:** Coordenação do cuidado: tentativas de superação das adversidades comunicacionais no fluxo assistencial fragmentado.

COMPONENTE TEMÁTICO	EXTRATOS DE FALAS DOS ENTREVISTADOS
ACS intermediava as informações clínicas entre usuários e equipe	“*Só ficamos sabendo* [retorno da consulta com especialista] *através do paciente, ou através do agente de saúde que mandamos ir atrás para saber o que foi que aconteceu*” (Monte Alegre, Piauí, Matopiba)
	“*Depois que o paciente sai da internação tem o acompanhamento, os ACS sempre me avisam*” (Jacareacanga, Pará, Norte Estradas)
Ausência de contrarreferência	“*Mas tipo, referência e contrarreferência, a gente não tem! Nem daqui* [próprio município], *nem de fora* [do município]” (Ipupiara, Bahia, Semiárido)
	“*Para a gente conseguir essa comunicação a gente precisa falar com o pessoal da secretaria para ver se eles conseguem entrar em contato com o hospital ou com a clínica para falar com esse profissional e ele dar um retorno. Na maioria das vezes, a gente fala com o familiar do paciente, é difícil a gente conseguir informação do próprio estabelecimento*” (Morpará, Bahia, Semiárido)
Usuários eram os porta-vozes da conduta terapêutica desenvolvida fora da UBS	“*Não* [sobre ter retorno do especialista], *é o relato do paciente*” (Vitória do Jari, Amapá, Norte Águas)
	“*As informações* [após consulta com especialista] *vêm pelo próprio paciente mesmo*” (Vila Bela da Santíssima Trindade, Mato Grosso, Vetor Centro-Oeste)
	“*Nós ficamos sabendo, porque acabamos ligando para o paciente. O paciente liga para nós, entra em contato*” (Monte Alegre, Piauí, Matopiba)
	“*Não acontece* [contato com o especialista], *a partir do momento que o TFD liberou* [para tratamento fora], *já é por conta do especialista. A gente só consegue saber do paciente quando ele volta para o município*” (Jacareacanga, Pará, Norte Estradas)
Enfermeiros restabeleciam o vínculo comunicacional e viabilizavam a continuidade do cuidado	“...*a gente conhece todo mundo, no dia que aquela pessoa não vem, quando eu vejo ela na rua, e ela não foi no grupão* [atividade coletiva], *eu já falo: ‘a senhora não foi no grupão!’ Então, a gente já sabe quem faltou, eu já conheço pelo nome. ‘A senhora não veio, mês que vem eu quero a senhora lá’. Assim, eles acabam voltando ele para mim*” (Rurópolis, Pará, Norte Estradas)
	“*Meu WhatsApp tem acho que mais de mil contatos: a região, o município. Todo mundo tem meu telefone! Tudo que precisa, mandam mensagens. Todo mundo tem* [meu celular], *pra tudo o que precisa*” (Rubelita, Minas Gerais, Norte Minas)
Enfermeiros realizavam contatos informais com especialistas ou utilizavam seu prestígio para contornar adversidades no fluxo assistencial	“*Eu não tenho diretamente* [com médico especialista], *tenho contato com o secretário e com a coordenadora da APS. Tem casos que como não vai dar pra esperar por regulação, então, eu ‘futuco’ o secretário pra ele dar ajuda de custo e fazer no particular. Nem todos os casos dá pra esperar, né?*” (Tasso Fragoso, Maranhão, Matopiba)
	“...*a gente vê dentro do nosso suporte, e eu entro em contato com a secretaria e vejo dentro da secretaria o que eles podem fazer para pular essa espera. Porque, às vezes, a gente acorda com um e articula com outro e consegue para os pacientes que estão com grande necessidade*” (Morpará, Bahia, Semiárido)
Enfermeiros buscavam alguma coordenação do cuidado com táticas para compor o mosaico de informações	“*A gente tenta ir atrás dessa informação sempre e não perder esse paciente aqui da unidade, a menos que ele não possa ficar dentro da cobertura. Mas a gente não tem uma resposta do serviço, a gente tem que ir atrás dessa informação, pois a comunicação não é feita de forma efetiva. Porque, a gente tem que ficar indo atrás o tempo todo, muitas vezes, só sabemos por meio das famílias mesmo*” (Morpará, Bahia, Semiárido)

ACS: agentes comunitários de saúde; APS: atenção primária à saúde; TFD: tratamento fora de domicílio; UBS: unidades básicas de saúde.

**Quadro 4:** Competência cultural residual: saber-fazer biomédico hegemônico em detrimento à diversidade cultural e étnica nos territórios.

COMPONENTE TEMÁTICO	EXTRATOS DE FALAS DOS ENTREVISTADOS
Práticas populares de cuidado, as terapias tradicionais e os praticantes leigos de cura estavam sempre presentes nos territórios dos municípios rurais remotos	“Garrafada (...). *Às vezes assim, num aborto espontâneo, algumas mulheres ‘Ah, eu não vou lá* [na UBS] *ver se precisa de fazer curetagem, não, eu vou tomar uma garrafada e limpa tudo’. Entendeu? A gente já teve uma situação em que a paciente que sofreu um aborto espontâneo precisava fazer uma curetagem, mas ela não retornou* [ao hospital]. *Tomou garrafada! Ela ficou bem, mas é uma questão de tradição. No mato* [zona rural] *mesmo, a população bugre tem as parteiras*” (Vila Bela da Santíssima Trindade, Mato Grosso, Vetor Centro-Oeste)
	“*Garrafada tem muito na zona rural. Direto eles vêm e falam: ‘Ah, tomei uma garrafada’. Principalmente as mulheres na questão ginecológica*” (Nova Lacerda, Mato Grosso, Vetor Centro-Oeste)
	“*A parteira é a que eu mais presencio, uso de garrafada aqui é para nossa região eles têm essa cultura muito forte aí eles usam muito essas garrafadas eles acreditam muito nesse lado mas naturalistas, outros mais assim da cultura mesmo da religião de feitiçaria Até mesmo porque como eu já falei, nós temos áreas indígenas que a cultura deles é muito forte Então existe aqui na nossa comunidade. A gente respeita e a gente consegue trabalhar bem mesmo com a cultura deles, passa as orientações sempre respeitando a cultura deles e fica a critério deles a escolha, quem prefira ter bebê em casa com parteira, mais elas vem fazer o pré-natal aqui, a gente passa todas as orientações fala que o adequado seria no hospital onde tem mais suporte em caso de intercorrência e tudo mais então elas fazem o parto domiciliar sendo bem consciente, respeitando a cultura delas porque a gente não pode ir bater de frente com algo tão forte que a cultura*” (Aveiro, Pará, Norte Águas)
	“*Temos muitas parteiras por aí. Eu aconselho as gestantes a irem para Vitória* [do Jari] *umas duas semanas antes da previsão do parto, mas muitas preferem parir em casa, por terem uma parteira próxima*” (Vitória do Jari, Amapá, Norte Águas)
	“*É mais na área indígena. Por exemplo, teve um caso de paciente que estava com cirrose hepática, aí, veio o povo todo da aldeia para fazer esses negócios de banho e dança; é difícil acontecer aqui na cidade ou nessas comunidades que não são indígenas*” (Jacareacanga, Pará, Norte Estradas)
Ausência de interlocução entre o saber popular e as ações biomédicas	“*Não há nenhum trabalho tradicional, nem UBS adaptadas para os quilombolas e ribeirinhos.* (...). *Temos pouco espaço para a parte cultural. Nosso cronograma é muito amarrado. A equipe é muito sobrecarregada*” (Redenção da Gurguéia, Piauí, Matopiba)
	“*Tem os curandeiros, mas eu não tenho contato com esse povo não, só ouço falar*” (Rurópolis, Pará, Norte Águas)
	“*Não* [tem articulação]. *Eu nem conheço. Só escuto falar: ‘Ah, mandou rezar o menino e o menino sarou. O menino tá ótimo’. Eu não entro nesse detalhe não, não conheço as pessoas que fazem esse tipo de reza*” (Rubelita, Minas Gerais, Norte de Minas)
Interculturalidade revelou-se em situações singulares	“*É que agora que deu uma parada, mas aqui, nós reunimos e levamos* [todas as parteiras do município] *para uma capacitação e troca de experiências. Nós já passamos 4 dias trocando experiências só com as parteiras tradicionais*” (Assis Brasil, Acre, Norte Estradas)
	“...*só tenho um ACS que fala Munduruku* [língua indígena], *aí, quando estou em dúvida, pois, às vezes, não entendo a indígena e ela não me entende também, aí, chamo ele* [o ACS]” (Jacareacanga, Pará, Norte Estradas)
	“...*ele* [usuário] *chega falando que já foi com o fulano de tal e tomou uma garrafada top, mas vamos fazer um tratamento terapêutico com medicação, né? A parteira é a nossa técnica em enfermagem, que ela já fez uns 300 partos aqui.* (...) *ela fez curso*” (Rurópolis, Pará, Norte Estradas)
	“*Nós temos inclusive, um grupo que já está trabalhando conosco.* [Grupo] *das Parteiras Tradicionais. Toda vez que tem alguma ação em educação em saúde, elas vêm, elas são convidadas. Tipo, Semana do Aleitamento Materno, Semana da Mortalidade Materna, Semana do Colo de Útero, agora, elas sempre são convidadas*” (Boa Vista de Ramos, Amazonas, Norte Águas)
	“*Temos muitas garrafadas, curandeiros, parteiras,* (...) *as pessoas fazem uso, eu pude perceber puxador, rezadeiros. A nossa técnica de enfermagem também é parteira!*” (Curuá, Pará, Norte Águas)

ACS: agentes comunitários de saúde; PSF: Programa Saúde da Família; UBS: unidades básicas de saúde.

Na sequência, realizou-se o cotejamento entre os componentes temáticos que, ao final, engendraram quatro categorias empíricas: (1) Protagonismo gerencial das enfermeiras nos territórios: porta de entrada e organização da agenda; (2) Abrangência e longitudinalidade das práticas da enfermeira: ações compartimentalizadas e centradas na queixa-conduta; (3) Coordenação do cuidado: tentativas de superação das adversidades comunicacionais no fluxo assistencial fragmentado; e (4) Competência cultural residual: saber-fazer biomédico hegemônico em detrimento da diversidade cultural e étnica nos territórios. 

O conjunto de dados contém as características essenciais do universo pretendido e seus resultados agregam elementos representativos e passíveis de transferibilidade ^32^ em outros cenários nacionais similares. 

A pesquisa foi aprovada pelo Comitê de Ética em Pesquisa, Escola Nacional de Saúde Pública Sergio Arouca, Fundação Oswaldo Cruz (Ensp/Fiocruz, parecer nº 2.832.559), com anuência dos municípios. Todos os participantes assinaram um Termo de Consentimento Livre e Esclarecido.

## Resultados e discussão 

A formulação das quatro categorias empíricas fundamentou-se nos resultados emergentes dos 34 componentes temáticos identificados (Quadros 1, 2, 3 e 4). A discussão está ancorada na concepção da APS como principal porta de entrada para produção do cuidado ^33^. Para a nomeação das categorias, foram considerados os atributos de uma APS robusta − acesso/primeiro contato, longitudinalidade, abrangência das práticas, coordenação do cuidado, orientação comunitária e competência cultural − que funcionaram como eixos analíticos orientadores da interpretação dos dados.

Buscou-se articular os atributos da APS com a prática das enfermeiras e a organização do seu processo de trabalho no contexto de municípios rurais remotos. Para tal, adotou-se como referencial teórico central o modelo proposto por Mendes-Gonçalves ^31^, o qual oferece subsídios para a análise crítica do processo de trabalho em saúde. Esse referencial foi complementado por aportes teóricos de autores que abordam as práticas em saúde sob uma perspectiva contra-hegemônica. Destaca-se a contribuição de Peduzzi et al. ^28^, no que tange à compreensão das práticas interprofissionais e à construção do trabalho em equipe; de Pinheiro ^23^, ao abordar o cuidado em saúde em sua dimensão ético-política; e de Campos et al. ^27^, ao propor os conceitos de clínica ampliada e cogestão como estratégias para repensar o trabalho em saúde, integrando autonomia profissional e responsabilidade coletiva.

Por fim, foram selecionados fragmentos representativos de relatos das enfermeiras de diferentes municípios rurais remotos e distintos *clusters* para ilustrar as categorias empíricas (Quadros 1, 2, 3 e 4).

### Protagonismo gerencial das enfermeiras nos territórios: porta de entrada e organização da agenda

Em todos os *clusters* estudados, garantir o acesso oportuno e facilitado às UBS tem sido um grande desafio às EqSF, notadamente em localidades mais afastadas das sedes municipais ^5^. Nesses cenários, as enfermeiras destacaram-se, com poucas diferenças/especificidades entre os *clusters*, por desenvolverem um escopo variado de competências em atividades gerenciais, práticas assistenciais individuais − em consultório e domicílio − e ações no território. Ainda assim, as atividades gerenciais e assistenciais em diferentes modelos de APS, embora diversificadas, revelam, igualmente, as vicissitudes das enfermeiras para compatibilizar as práticas individuais e coletivas ^9^, bem como, expõem a complexidade do trabalho da enfermeira em diferentes espaços sociais e para distintos ciclos de vida ou condições de saúde ^34^.

Diante da diversidade dos contextos, as equipes de APS organizavam suas ações de forma adaptada à dinâmica social de cada território. De modo convergente, a UBS se consolidou como principal espaço de busca por cuidado, funcionando, em geral, em horário comercial. Observou-se, ainda, que a organização dos fluxos administrativos, assistenciais e das práticas comunitárias era majoritariamente centralizada nas enfermeiras.

À vista disso, as EqSF, embora constituídas por diferentes profissionais, concentram as atividades gerenciais na enfermeira que, por sua vez, acumula múltiplas práticas de gerenciamento e de assistência ^35^, ainda que devam ser exercidas de modo compartilhado e horizontal combinando responsabilidade e autonomia. A autoridade gerencial e o protagonismo assistencial da enfermeira em diferentes cenários são importantes atributos da prática ^36^, o que torna tal profissão requisitada pelos municípios para assumir espaços na gestão local ^37^, conquanto persistam lacunas na formação que atenda a essa competência ^38^. A incongruência reside na sobrecarga laboral, estresse ocupacional ^39^ e pouco reconhecimento por meio de salários e outros direitos trabalhistas ^40^.

A agenda era organizada, predominantemente, por ações programáticas segmentadas por ciclo de vida ou condição de saúde, por meio de uma “semana típica” que orientava o conjunto de ações assistenciais e os grupos populacionais atendidos em cada turno. Após consultas programáticas com médicos ou enfermeiras, especialmente em casos de agravos crônicos, alguns usuários saíam da UBS com retorno agendado − prática não sistemática e marcada por variações na organização das agendas. Em equipes com menor flexibilidade e foco estrito em ações programáticas, ainda que de forma pontual, observou-se que a agenda se tornava uma barreira de acesso, ao não contemplar as demandas espontâneas dos usuários.

Em contraste, havia equipes em que o atendimento por demanda espontânea era predominante ou mesmo exclusivo, especialmente nas ações itinerantes em territórios distantes da UBS. Em outras, essa modalidade coexistia com a oferta programada ou ganhava centralidade diante da ausência de profissionais, notadamente médicos. Algumas equipes, mesmo com agendas estruturadas, demonstravam sensibilidade às especificidades das populações rurais, sobretudo as mais isoladas ou com dificuldade de transporte. Nesses casos, priorizavam os atendimentos no turno da manhã, concentravam-nos nos dias com maior disponibilidade de transporte ou buscavam encaixá-los na programação, evitando que os usuários retornassem sem acesso ao cuidado.

Práticas centradas nas pessoas requerem um balanço entre atendimentos às demandas programadas e à demanda espontânea, corroborando uma APS que seja resolutiva e se proponha a ser reconhecida como serviço de procura regular ^10^. A organização da oferta e da demanda atenta à dinâmica do território distingue a APS integral, resolutiva e comunitária de outras modalidades que realizam ações programáticas concentradas em certos grupos populacionais, que excluem algumas pessoas e limitam seu acesso. A organização do processo de trabalho de EqSF por meio de agendas muito fechadas, que somente preveem atendimento à demanda organizada, limita o acesso oportuno, por outra via, unidade de saúde da família que somente atende à demanda espontânea incorre no risco de centrar o cuidado em modelo tradicional de queixa-consulta ^10^.

Nos municípios rurais remotos, em decorrência das distâncias e dificuldades de acesso à UBS, para populações mais dispersas em áreas rurais, o atendimento era itinerante, com visitas esporádicas da equipe a diferentes comunidades e atendimento em locais adaptados. Pela falta de modelos mais específicos de atenção à saúde rural no Brasil, esses atendimentos, muitas vezes, ocorrem em locais adaptados, sem condições ideais ^17^. Em alguns municípios, há pequenas unidades que funcionam como postos avançados, nos quais, em geral, técnicas de enfermagem atendem regularmente a população em seu escopo de prática e para onde a EqSF se desloca em intervalos variados. Nesses casos, a demanda era organizada pelos ACS ou, na ausência desses, por algum líder comunitário. Por fim, o ACS, também, é um importante sujeito discricionário da oferta, pois direciona às UBS um conjunto de usuários, conforme captação nos territórios, ou seja, regulando a oferta e a demanda às equipes ^41^.

A existência de áreas descobertas influenciava negativamente na oferta de uma agenda programática, pois o fluxo de usuários ficava confuso sem a presença do ACS no território para articular os aprazamentos dos grupos prioritários, contribuindo para busca espontânea na UBS. Outros desafios da agenda na UBS foram as características territoriais, uma vez que, especialmente na região norte, o ciclo das águas determina o fluxo dos usuários nas UBS que, por sua vez, requer ajustes periódicos na organização das ações ^18^. Ademais, a rotatividade de profissionais e a restrição de transporte para deslocamento da população rural à UBS impunham rearranjos na agenda dos serviços, aspectos, esses, amplamente documentados em diferentes estudos ^5^ ^,^ ^42^.

Todos esses empecilhos e incertezas no atendimento em local adequado e tempo oportuno contribuem para uso inapropriado dos serviços, elevado tempo de espera e, consequentemente, insatisfação com a APS ^43^. Estudos sugerem que diversificar os modos de marcação de consultas − por telefone, e-mail e recursos digitais/aplicativos ^44^ −, acolher a demanda espontânea, expandir o escopo de práticas das enfermeiras ^12^, incluir consultas remotas ^45^, realizar atividades assistenciais itinerantes ^46^ e ampliar as ações em grupo no território ^47^ são estratégias capazes de ampliar e diversificar o acesso em municípios e áreas dispersas. 

Independentemente dos obstáculos impostos ao atendimento na UBS, em todos os municípios rurais remotos, sobressaiu-se a articulação das enfermeiras na busca por contornar as adversidades, restabelecer fluxos para os usuários e minimizar ao máximo a desassistência. Inclusive, havia enfermeiras que residiam na zona rural e ficavam à disposição para atender/encaminhar as intercorrências fora do expediente, bem como estavam fortemente vinculadas à comunidade. As características da ruralidade impõem importantes desafios organizacionais, ainda assim, enfermeiras das áreas rurais têm intensa relação com a população e, por isso, respondem de maneira criativa e implicada às demandas da comunidade, ou seja, concebem o cuidado como um valor ético-político para consecução de práticas integrais ^23^.

### Abrangência e longitudinalidade das práticas da enfermeira: ações compartimentalizadas e centradas na queixa-conduta

De modo geral, alguns aspectos contribuíam para que as práticas profissionais tivessem um caráter mais individual do que compartilhado nas equipes dos municípios rurais remotos. Assim, a rotatividade, especificamente de médicos, comprometia a longitudinalidade do cuidado e havia um constante recomeço (retrabalho) na conduta terapêutica entre profissional-paciente. Igualmente, os períodos com equipes desfalcadas geravam demandas acumuladas na população que, por sua vez, buscava a UBS por demanda espontânea, contribuindo para a predominância de práticas na lógica da queixa-conduta. 

As características dos territórios também impunham desafios ao trabalho compartilhado. Em áreas remotas, com dificuldades de acesso ou transporte à UBS, usuários de regiões mais dispersas eram frequentemente atendidos por demanda espontânea, inseridos de forma pontual na rotina programática. Diante desse cenário, enfermeiras e médicos adotavam práticas mais isoladas e compartimentalizadas, visando agilizar os atendimentos e dar conta da elevada demanda assistencial.

Em municípios rurais remotos, um mosaico de problemas, agindo em sinergia, torna premente o processo de trabalho colaborativo na APS ^48^ e, consequentemente, requer maior engajamento das equipes numa perspectiva interprofissional ^28^. Paradoxalmente, a escassez, a rotatividade e o perfil predominante de médicos que atuam nestes contextos ^49^ comprometem a assunção de um trabalho compartilhado com a equipe em decorrência, inclusive, da própria divisão social do trabalho ^50^ e das condições laborais da força de trabalho da enfermeira ^24^. 

As barreiras de acesso ^8^ − políticas, geográficas, socioeconômicas, técnico-organizacionais e simbólicas − às UBS em municípios rurais remotos, também desfavorecem o trabalho colaborativo, sobretudo pela produção de demanda reprimida e o tensionamento por um atendimento centrado na doença e, por conseguinte, na medicalização social ^51^. Por meio desse modelo, o compartilhamento das práticas restringe-se a um agir instrumental pautado na racionalidade técnico-científica de cada profissional, prejudicando a construção de acordos mútuos entre profissionais e destes com os usuários e famílias ^28^.

Outrossim, não havia um padrão comum para todas as equipes em relação ao trabalho compartilhado entre enfermeira e outros membros da equipe. De forma geral, o compartilhamento dava-se por atendimento intercalado entre médico e enfermeira, por meio de revezamento na conduta clínica, frequente no cuidado materno-infantil. Ainda nesse aspecto, em algumas equipes, o trabalho da enfermeira ficava submisso ao apoio à prática médica ou sujeito à sua avaliação pós-consulta. Em outras equipes, a consulta individual geral era realizada pelo médico, enquanto a enfermeira realizava as ações programáticas, assumia as práticas preteridas pelos médicos (comumente, o exame preventivo, as ações comunitárias e as atividades gerenciais) ou, simplesmente, limitava-se à divisão de tarefas sem uma ação, verdadeiramente comum. 

Em algumas UBS, os casos crônicos controlados eram acompanhados pela enfermeira, enquanto os descompensados eram direcionados ao médico. Embora menos frequentes, ocorriam consultas sequenciais no mesmo dia ou atendimentos conjuntos em situações clínicas específicas que exigiam avaliação compartilhada, ainda que sem construção de plano terapêutico comum. Tais práticas respondiam a necessidades individuais − clínicas e/ou sociais − e não configuravam um padrão no processo de trabalho das equipes.

Os resultados expõem, embora de maneira pontual, um trabalho em equipe numa perspectiva multiprofissional colaborativa entre médicos e enfermeiras, uma vez que as ações compartimentalizadas e centradas na queixa-conduta dominam o processo de trabalho nas UBS. Em contrapartida, enfermeiras têm incorporado tarefas antes exclusivas à categoria médica, sobretudo de diagnóstico e terapêutica, respaldadas por protocolos, ampliando o escopo de práticas com novas atribuições (*task-shifting*) ^42^ ^,^ ^52^. Globalmente, reconhece-se que as enfermeiras possuem um perfil profissional que converge com aquele exigido para atuação em territórios rurais, uma vez que exercem um rol amplo de práticas em assistência, gestão e educação. Não obstante, sua plena capacidade sofre reiteradas restrições pela baixa compreensão da contribuição e do processo de trabalho da enfermeira, havendo centralização excessiva da oferta de atenção à saúde sob domínio dos médicos ^42^ ^,^ ^49^. De tal modo, é indispensável fortalecer e reconhecer a autonomia da enfermeira, junto com uma cultura de colaboração interprofissional.

Ademais, as reuniões de equipe, convocadas e presididas pelas enfermeiras, serviam como espaço de interlocução para compartilhamento de agendas, rotinas e, esporadicamente, discussão de algum caso clínico mais emblemático, embora com menor participação de médicos. De modo geral, as enfermeiras estavam envolvidas direta ou indiretamente com todas as ações individuais e coletivas na UBS ou na comunidade, convergindo em um padrão de colaboração intrínseco às práticas multiprofissionais em todos os municípios rurais remotos. Em muitos casos, o trabalho compartilhado restringia-se às ações de educação em saúde para grupos específicos, especialmente em formato de palestras na comunidade, envolvendo enfermeiras, agentes comunitários de saúde (ACS) e equipe do Núcleo de Apoio à Saúde da Família (NASF). Por fim, chama a atenção que as práticas desenvolvidas, independentemente da interface entre os profissionais e o seu grau de compartilhamento, não se mostravam articuladas com a população nas decisões sobre sua saúde. 

Em síntese, havia interação e comunicação entre profissionais de diferentes áreas nas equipes, sendo este um dos atributos centrais e uma condição *sine qua non* para o trabalho interprofissional ^28^. Contudo, o protagonismo do processo de trabalho de enfermeiras em municípios rurais remotos revela-se, também, na estreita dependência da organização da agenda e na definição dos fluxos dos diferentes profissionais nas UBS. Em certa medida, a gerência das UBS atribuída às enfermeiras em todas os municípios rurais remotos, contraditoriamente, confere-lhes um status de poder relativo sobre a programação/planejamento das ações na APS ^11^, porém, ao mesmo tempo, representa uma indisponibilidade/recusa dos demais profissionais, especialmente médicos, em assumirem tal responsabilidade organizacional ^10^ ^,^ ^49^. Além disso, as enfermeiras recebem menores salários, comumente cumprem 40 horas semanais de trabalho nas UBS e acumulam um leque diverso de atividades gerais − comum à equipe − e específicas do seu núcleo profissional ^9^. Tais constatações teóricas e empíricas ressaltam a precarização do trabalho da enfermeira em detrimento à sua importância na APS ^24^ ^,^ ^25^. 

### Coordenação do cuidado: tentativas de superação das adversidades comunicacionais no fluxo assistencial fragmentado

Nos municípios rurais remotos, as informações necessárias à coordenação do cuidado de usuários que foram assistidos em serviços fora da APS, mesmo que encaminhados pela UBS, eram repassadas à equipe, frequentemente, pelos ACS, principalmente pela ausência de contrarreferência ou qualquer outra formalização comunicacional entre os diferentes níveis de atenção. Nesse aspecto, os ACS, frequentemente, intermediavam a visita domiciliar após a alta hospitalar, para que médicos ou enfermeiras assistissem o usuário que não podia ir à UBS. Os ACS destacam-se em distintas experiências como um trabalhador imprescindível para a comunicação entre serviços e comunidade ^53^, inclusive, para viabilizar as informações sobre o fluxo assistencial e alguma coordenação horizontal pela equipe em áreas rurais ^54^. Ademais, as enfermeiras buscavam diretamente com os usuários as informações sobre a conduta terapêutica realizada fora do âmbito da APS, muitas vezes, por busca ativa no território. 

Desse modo, as enfermeiras, em todos os municípios rurais remotos, realizavam um trabalho fundamental para restabelecer o vínculo comunicacional e viabilizar a continuidade do cuidado das pessoas que necessitavam de alguma assistência fora da APS. Fundamentalmente, eram o elo entre os usuários e as centrais de marcação de consultas e, comumente, buscavam intervir para agilizar o fluxo, especialmente, para os casos que requeriam atenção clínica diferenciada. Dessa forma, enfermeiras realizavam contatos informais com médicos especialistas da rede ou utilizavam seu prestígio no município para contornar adversidades no fluxo assistencial e tentar diminuir o tempo de espera. Os resultados ratificam outros estudos que destacam a atuação das enfermeiras na garantia de coordenação em função do envolvimento mais próximo aos usuários e suas famílias, protagonismo na resolução e manejo de cuidados na APS por meio das consultas de enfermagem, utilização de prontuários eletrônicos, ações de educação permanente em saúde, atuação direta no processo de referência e contrarreferência entre os serviços da Rede de Atenção à Saúde (RAS), além do acompanhamento pós-alta, por meio da comunicação e articulação com profissionais e serviços de saúde ^55^. Nesse sentido, as práticas das enfermeiras permitem, de alguma forma, concretizar a coordenação na perspectiva do sistema de saúde e a continuidade na experiência dos usuários ^55^.

Habitualmente, as enfermeiras ou médicos da APS não tinham contato com os profissionais da atenção especializada ou hospitalar, requerendo que os usuários fossem os porta-vozes da conduta terapêutica desenvolvida fora da UBS. Embora pacientes e familiares sejam frequentemente alçados ao posto de único elo comunicacional entre os pontos de atenção na RAS com a missão de facilitar ou produzir alguma continuidade assistencial, tal responsabilidade não vem acompanhada das habilidades ou confiança necessárias. A repetição das histórias clínicas e pessoais aumenta a sensação de insegurança e dificulta a navegação pelo sistema de saúde ^56^. Frente a este problema, as enfermeiras buscavam algum nível de coordenação do cuidado e lançavam mão de táticas para compor o mosaico de informações necessárias à continuidade assistencial: busca ativa do usuário; reunião de documentos (receitas e resultados de exames) em posse do paciente/familiar; interlocução com a secretaria de saúde e, quando possível, tentativa de contato com o profissional/serviço que atendeu o usuário. 

Em caso das enfermeiras que residiam na comunidade rural, havia um envolvimento estreito com as demandas dos usuários e, nesse sentido, realizavam alguma coordenação do cuidado − agendando transporte sanitário, encaminhando documentos para marcação de consultas/exames, interferindo nos fluxos, buscando articular as informações clínicas etc. − por meio do contato direto com os pacientes pelo celular. 

### Competência cultural residual: saber-fazer biomédico hegemônico em detrimento à diversidade cultural e étnica nos territórios

Em todas as entrevistas, em menor ou maior amplitude, as práticas populares de cuidado, as terapias tradicionais e os praticantes leigos de cura estavam presentes no cotidiano dos territórios dos municípios rurais remotos. Ainda assim, de forma unânime, nenhuma enfermeira estabelecia uma interlocução entre o saber popular e as ações biomédicas das práticas estabelecidas na APS, ainda que reconhecessem a diversidade cultural e étnica que povoava os territórios. 

A competência cultural é um importante atributo da APS, por meio da qual a equipe deve reconhecer as diferentes necessidades dos grupos populacionais, suas características étnicas, raciais e culturais, entendendo suas representações dos processos saúde-enfermidade-cuidado. A ausência de competência cultural reforça, especialmente, em territórios com povos e comunidades tradicionais, (ribeirinhos, quilombolas, população indígena etc.), como em municípios rurais remotos, as iniquidades em saúde. Ainda assim, a competência cultural não deve ser tomada como uma ação instrumental da prática cotidiana voltada à intervenção biomédica, mas uma estratégia de compromisso ético-político com o cuidado sensível e intercultural ^13^.

Outrossim, a interculturalidade manifestou-se em situações pontuais, como um curso para parteiras na Região Norte e o uso pessoal de terapias populares como a garrafada, por enfermeiras, embora não integradas à prática profissional. Em um dos territórios do norte, uma técnica de enfermagem também atuava como parteira reconhecida pela comunidade, ainda que sua atuação não estivesse vinculada às ações da APS. Além disso, no semiárido baiano, uma enfermeira residente na zona rural participava da pastoral católica e do movimento dos trabalhadores rurais, fortemente influenciada pela trajetória militante de sua mãe em movimentos populares no território.

A excepcionalidade nas práticas interculturais das enfermeiras, em municípios rurais remotos, restrita ao uso pessoal/familiar, reforça a hegemonia do saber biomédico e a proeminência de ações individuais e coletivas na APS sem interlocução com os saberes e fazeres comunitários. Entretanto, trata-se de um obstáculo que extrapola as escolhas individuais dos profissionais, pois estão presentes, até mesmo, na formulação de políticas de saúde que reproduzem o caráter etnocêntrico com inúmeras contradições e negligências que não contemplam, de fato, o intercâmbio e a articulação com o saber tradicional e outras cosmovisões acerca dos processos de saúde-doença-cuidado-morte ^57^. A interculturalidade, por outro lado, é um importante enfoque ético-político para a construção de práticas integrais na APS ^58^, sendo, cada vez mais, parte das políticas sanitárias de países latino-americanos, embora com distintos níveis de implementação ^59^.

Ademais, as enfermeiras, junto às equipes, organizavam agendas, ações e intervenções considerando adversidades como dificuldades de trafegabilidade, escassez de transporte à UBS, condições climáticas e os fluxos cotidianos da população. Ainda que de forma pontual, essas práticas demonstravam certa competência cultural e evidenciavam a importância da APS territorializada. Assim, mesmo diante de limitações, as enfermeiras nos municípios rurais remotos buscavam contornar obstáculos e desenvolver práticas sensíveis às demandas das pessoas e das comunidades.

## Considerações finais

A análise da atuação e do compartilhamento das práticas das enfermeiras na municípios rurais remotos brasileira revela seu protagonismo na gestão das UBS e equipes de APS, especialmente nas atividades que viabilizam ações gerenciais, assistenciais e comunitárias. Simultaneamente, trabalham de modo estratégico na coordenação do cuidado e na comunicação entre os pontos de atenção e os usuários, em contextos marcados pela rotatividade médica e por barreiras geográficas significativas de acesso à RAS.

O predomínio de processos de trabalho compartimentalizados, ancorados no modelo assistencial queixa-conduta, com frágil atuação interprofissional e limitada competência cultural, constitui um desafio à produção do cuidado na APS, devendo ser analisada à luz da dinâmica das equipes. Por fim, considerando o protagonismo das enfermeiras, especialmente nas EqSF em municípios rurais remotos, defende-se que sua valorização ocorra não apenas por meio de formação e remuneração condizentes, com garantia do piso nacional, mas também mediante políticas sensíveis a arranjos que ampliem sua inserção em contextos específicos. 

Como limitação do estudo, destaca-se o fato de que os dados foram produzidos exclusivamente a partir de entrevistas com enfermeiras, restringindo o contraste com a experiência e a percepção de outros sujeitos sociais.
